# The CORM ALF-186 Mediates Anti-Apoptotic Signaling via an Activation of the p38 MAPK after Ischemia and Reperfusion Injury in Retinal Ganglion Cells

**DOI:** 10.1371/journal.pone.0165182

**Published:** 2016-10-20

**Authors:** Felix Ulbrich, Kai B. Kaufmann, Alexander Meske, Wolf A. Lagrèze, Michael Augustynik, Hartmut Buerkle, Carlos C. Ramao, Julia Biermann, Ulrich Goebel

**Affiliations:** 1 Department of Anesthesiology and Critical Care, Medical Center – University of Freiburg, Faculty of Medicine, University of Freiburg, Freiburg, Germany; 2 Eye Center, Medical Center – University of Freiburg, Faculty of Medicine, University of Freiburg, Freiburg, Germany; 3 Instituto de Tecnologia Química e Biológica-António Xavier, Universidade Nova de Lisboa, Oeiras, Portugal; 4 Alfama Ltd., Instituto de Biologia Experimental e Tecnológica, IBET, Oeiras, Portugal; Indian Institute of Science Education and Research, INDIA

## Abstract

**Purpose:**

Ischemia and reperfusion injury may induce apoptosis and lead to sustained tissue damage and loss of function, especially in neuronal organs. While carbon monoxide is known to exert protective effects after various harmful events, the mechanism of carbon monoxide releasing molecules in neuronal tissue has not been investigated yet. We hypothesize that the carbon monoxide releasing molecule (CORM) ALF-186, administered after neuronal ischemia-reperfusion injury (IRI), counteracts retinal apoptosis and its involved signaling pathways and consecutively reduces neuronal tissue damage.

**Methods:**

IRI was performed in rat´s retinae for 1 hour. The water-soluble CORM ALF-186 (10 mg/kg) was administered intravenously via a tail vein after reperfusion. After 24 and 48 hours, retinal tissue was harvested to analyze mRNA and protein expression of Bcl-2, Bax, Caspase-3, ERK1/2, p38 and JNK. Densities of fluorogold pre-labeled retinal ganglion cells (RGC) were analyzed 7 days after IRI. Immunohistochemistry was performed on retinal cross sections.

**Results:**

ALF-186 significantly reduced IRI mediated loss of RGC. ALF-186 treatment differentially affected mitogen-activated protein kinases (MAPK) phosphorylation: ALF-186 activated p38 and suppressed ERK1/2 phosphorylation, while JNK remained unchanged. Furthermore, ALF-186 treatment affected mitochondrial apoptosis, decreasing pro-apoptotic Bax and Caspase-3-cleavage, but increasing anti-apoptotic Bcl-2. Inhibition of p38-MAPK using SB203580 reduced ALF-186 mediated anti-apoptotic effects.

**Conclusion:**

In this study, ALF-186 mediated substantial neuroprotection, affecting intracellular apoptotic signaling, mainly via MAPK p38. CORMs may thus represent a promising therapeutic alternative treating neuronal IRI.

## Introduction

As an organ with high demand of nutrients and oxygen, the human brain is very sensitive to hypoperfusion. In patients suffering from stroke, irreversible neuronal cell death may occur within minutes leading to substantial neuronal deficit and increased mortality.[1–4]

Similarly, retinal ischemia and reperfusion injury leads to neuronal cell death and plays an important role in the pathophysiology of several eye diseases such as diabetic retinopathy and glaucoma.[5, 6]

In the last years a multitude of substances have been tested pre-clinically regarding their neuroprotective potential.[7] However, all of them showed either a weak effect only, or failed to prove relevance in clinical practice at all.

In human organism, carbon monoxide (CO) is produced endogenously as one elimination product of heme oxygenase (HO), an enzyme that facilitates heme catabolism and catalyzes the degradation of heme to biliverdin, CO and ferrous ions.[8, 9] It has been shown that even exogenously applied CO may mediate protection in the context of ischemia and reperfusion. Although poisonous at high concentration, inhaled carbon monoxide has attracted attention as a potential therapeutic agent. Being administered after ischemia-reperfusion injury (IRI) and transplantation, CO appears to protect vital organs including heart, lung, kidney and liver.[10–13] To do so, CO interacts with a variety of physiological processes, among which some of them are responsible for apoptosis. As far as known, targets of CO action are the binding to soluble guanylate cyclase (sGC), stimulation of cGMP production, activation of ca^2+^-dependent potassium channels and stimulation of mitogen-activated protein kinases.[14]

Due to the fact of difficulties in handling a gaseous–and dose-dependently toxic–agent and the negative effects of inhaled CO on oxygen transport, molecules containing a heavy-metal framework were created as carriers of covalently-bound CO, defined as carbon monoxide releasing molecules (CORM). These substances, administered either orally or intravenously ensure a constant liberation of CO and delivered a predictable and controllable amount of CO to organs and tissue.[15] The carbon monoxide releasing molecule ALF-186 presents a special group of CORMs, which is water-soluble and releases its bound CO molecules in a slower manner than other CORMs do.[16]

Although described in some organ systems, therapeutic effects of CORMs on neuronal structures following neuronal damage have not been investigated so far. In this *in-vivo* study we analyzed the impact of the CORM ALF-186 after neuronal IRI and the underlying mechanism. We hypothesized that ALF-186 treatment mediates anti-apoptotic effects and protects retinal ganglion cells after IRI via the p38 MAPK. In future, CO might be a promising therapeutic option reducing neuronal damage.

## Materials and Methods

### Animals

**Fig 1:**
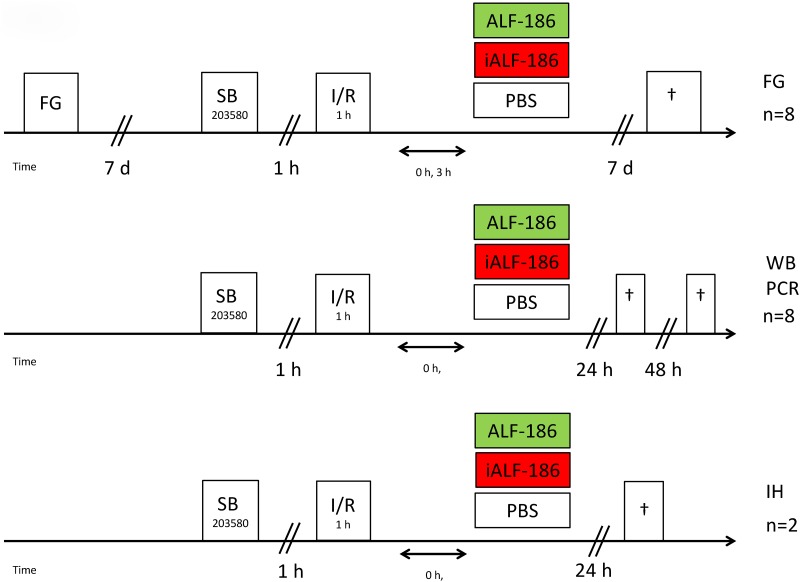
Time flow of the different experimental settings. First row: Fluorogold (FG) labeling seven days prior to IRI, subsequent treatment with or without p38 inhibitor (SB203580) and/or iALF-186/ALF-186 and enucleation another seven days later, giving FG the chance to promote into RGC via axonal transport. Second row: IRI with or without p38 inhibitor (SB203580) and/or iALF-186/ALF-186 treatment and enucleation 24 or 48 hours after IRI for molecular analysis. Third row: IRI with or without p38 inhibitor (SB203580) and/or iALF-186/ALF-186 treatment and enucleation 24 or 48 hours after IRI for immunohistochemical analysis.

Adult male and female Sprague-Dawley rats (1:1, 280-350g bodyweight, Charles River, Sulzfeld, Germany) were used in these experiments. Animals were fed with a standard diet *ad libitum*, being kept on a 12-h light/12-h dark cycle. All procedures involving the animals concurred with the statement of The Association for Research in Vision and Ophthalmology for the use of animals in research and were approved a priori by the Committee of Animal Care of the University of Freiburg in accordance with the ARRIVE guidelines (Permit No: 35–9185.81/G-11/81). All types of surgery and manipulations were performed under general anesthesia with isoflurane/O_2_ for retrograde labeling with fluorogold (FG) or with a mixture of intraperitoneally administered ketamine 50 mg/kg (Ceva-Sanofi, Duesseldorf, Germany) and xylazine 2 mg/kg (Ceva-Sanofi) for the ischemia-reperfusion experiment. Body temperature was maintained at 37°C ± 0.5°C with a heating pad. After surgery, buprenorphine (Temgesic^®^ 0.5 mg/kg; Essex Pharma, Solingen, Germany) was applied intraperitoneally to prevent pain. During recovery from anesthesia, the animals were placed in separate cages. The number of animals used for RGC quantification and molecular analysis was n = 8 per group. For analysis of mRNA- and protein-expression retinal tissue was harvested at t = 24 h and 48 h after reperfusion. (see flow-chart in Fig 1).

### Retrograde labeling of RGC

Sprague Dawley rats were anesthetized, placed in a stereotactic apparatus (Stoelting, Kiel, Germany) and retrograde RGC-labeling was done as described previously, briefly summarized as follows: The skin overlying the skull was cut open und retracted. The lambda and bregma sutures served as landmarks for drilling three holes on each site of the bregma sutures. A total amount of 7.8 μl fluorogold (FG) (Fluorochrome, Denver, CO, USA) dissolved in DMSO/PBS was injected into both superior colliculi through the drilling holes. To ensure adequate retrograde transport of FG into the RGC´s perikarya, further experimental interventions were done 7 days after retrograde labeling.

### Retinal ischemia/reperfusion injury and treatment with ALF-186

Following randomization, rats were sedated as previously described [17–19] and the anterior chamber of the left eye was cannulated with a 30-gauge needle connected to a reservoir containing 0.9% NaCl. Intraocular pressure was increased to 120 mmHg for 60 minutes and ocular ischemia was confirmed microscopically by interruption of the retinal circulation. Reperfusion was initiated by removing the needle tip promptly. Rats without immediate recovery of retinal perfusion at the end of the ischemic period or those with lens injuries were excluded from the study, since the latter prevents RGC death and promotes axonal regeneration. To evaluate possible neuroprotective effects of the treatment with carbon monoxide, released from ALF-186, animals were randomly assigned to receive either treatment with ALF-186 (10 mg/kg body weight i.v., dissolved in PBS) or PBS (vehicle control) alone. Both treatment options were injected intravenously, either immediately or with a delay of 3 hours after IRI. To exclude that the molybdenum-containing metal backbone of ALF-186 exerts protective or crucial effects itself, inactivated ALF-186 (iALF-186) was injected 24 hours after dissolving. In a later set of experiments, rats received the p38 MAPK inhibitor SB203580 (1 mg/kg bodyweight i.v., Sigma-Aldrich, #S8307) sixty minutes prior to IRI and ALF-186 treatment.

### RGC quantification

Animals were sacrificed by CO_2_-inhalation 7 days after ischemia. Retinal tissue was immediately harvested, placed in ice-cold Hank´s balanced salt solution and further processed for whole mount preparation. Retinae were carefully placed on a nitrocellulose membrane with the ganglion cell layer (GCL) on top. After removing the vitreous body, retinae were fixed in 4% paraformaldehyde for 1 h and then embedded in mounting media (Vectashield; Axxora, Loerrach, Germany). The densities of FG-positive RGC were determined in blinded fashion using a fluorescence microscope (AxioImager; Carl Zeiss, Jena, Germany) and the appropriate bandpass emission filter (FG: excitation/emission, 331/418 nm), as previously described. Briefly, we photographed 3 standard rectangular areas (0.200 mm x 0.200 mm = 0.04 mm^2^) at 1, 2 and 3 mm from the optic disc in the central regions of each retinal quadrant. Hence, we evaluated an area of 0.48 mm^2^ per retina. To calculate the average RGC density in cells/mm^2^, we multiplied the number of analyzed cells/0.04 mm^2^ with 25. Secondary FG stained activated microglia cells (AMC) after RGC phagocytosis were identified by morphologic criteria and excluded from calculation. All data are presented as mean RGC densities [cells/mm^2^] ± SD.

### Immunohistochemical staining

Immunohistochemistry was performed to identify p38 expressing cells in the retina 24 h after intervention. Rat eyes (n = 2 per group) were enucleated and immediately fixed in 4% paraformaldehyde overnight at 4°C. After washing in Dulbecco`s phosphate buffered saline before and after post-fixation in 30% sucrose for 5–6 h at 22°C, eyes were embedded in compound (Tissue-Tek; Sakura-Finetek, Torrance, CA, USA), and frozen in liquid nitrogen. Frozen sections (10 μm) were cut through the middle third of the eye and collected on gelatinized slides. Double immunolabeling was performed according to standardized protocols with monoclonal antibodies brain-specific homeobox/POU domain protein 3a Brn-3a (sc-390078; dilution 1:200; Santa Cruz Biotechnology; Dallas, TX, USA) with p38 (ab7952, dilution 1:200; Abcam, Cambridge, UK). Primary antibodies were then conjugated with their corresponding secondary antibody (red fluorescence: Alexa Fluor 647, mouse anti-rabbit, dilution 1:1000, Jackson ImmunoReaserch Europe, 211-605-109; green fluorescence: Cyanine Cy^™^2, donkey anti-goat, dilution 1:1000, Jackson ImmunoResearch Europe, 705-225-147). The nuclei of cells in the retina were stained with 4´,6-diamino-2-phenylindole dihydrochloride hydrate (DAPI, Sigma, Taufkirchen, Germany) added to the embedding medium (Mowiol; Calbiochem, San Diego, CA, USA). Slides were examined under a fluorescence microscope (Axiophot; Carl Zeiss, Jena, Germany).

### Western blot analysis

24 h and 48 h after ischemia retinal tissue for analysis of protein expression was harvested. Total protein from ¾ of retina was extracted and processed for Western Blot as described previously. The membranes were blocked with 5% skim milk in Tween20/PBS and incubated in the recommended dilution of protein specific antibody (p-ERK1/2 #4370, p-p38 #9211, p-JNK #9251, cleaved Caspase-3 #9664, Bax #2722, Bcl-2 #2876, Cell Signaling Technology, Danvers, MA, USA) overnight at 4°C. After incubation with a horseradish peroxidase-conjugated anti-rabbit secondary antibody (GE Healthcare, Freiburg, Germany), proteins were visualized using the ECL plus Chemiluminescence Kit (GE Healthcare). For normalization, blots were re-probed with ERK1/2 (#4695), p38 (#9212), JNK (#9258), Caspase-3 (#9665) and ß-Actin (#4967S, all Cell Signaling Technology, Danvers, MA, USA). Relative changes in protein expression in IR injured retinas either with or without ALF-186 were calculated in relation to the corresponding non-ischemic retinae.

### Real time polymerase chain reaction (RT-PCR)

From retinal tissue harvested 24 h and 48 h after ischemia, total RNA from ¼ of retina was extracted using a column-purification based kit (RNeasy Micro Kit, Qiagen, Hilden, Germany) according to the manufacturer´s instructions. Reverse transcription was performed with 50 ng of total RNA using random primers (High Capacity cDNA Reverse Transcription Kit, Applied Biosystems, Darmstadt, Germany). Real time polymerase chain reactions (RT-PCR) were done with TaqMan^®^ probe-based detection kit (TaqMan^®^ PCR universal mastermix, Applied Biosystems, Darmstadt, Germany). Following primers were used: Caspase-3 #Rn00563902_m1, Bax #Rn02532082_g1 and Bcl-2 #Rn99999125_m1 (all from Applied Biosystems, Darmstadt, Germany). The PCR assays were then performed on a RT-PCR System (ABI Prism 7000, Applied Biosystems, Darmstadt, Germany) with the following cycling conditions: 95°C for 10 min, 40 cycles of 95°C for 10 sec and 60°C for 1 min. Reaction specificity was confirmed by running appropriate negative controls. Cycle threshold (CT) values for each gene of interest were normalized to the corresponding CT values for GAPDH (ΔCT). Relative gene expression in IR injured retinal tissue either with injection of ALF-186 or PBS was calculated in relation to the corresponding gene expression in the non-injured retinal tissue of each individual animal (ΔΔCT).

### Statistical analysis

Data was analyzed using a computerized statistical program (SigmaPlot Version 11.0, Systat Software Inc., San Jose, CA, USA). The results are presented as means (±SD) after normal distribution of data had been verified. One-way ANOVA for repeated measurements was used for between-group comparisons with post hoc Holm-Sidak test and Kruskal Wallis test for data with lack of normal distribution. A p-value<0.05 was considered statistically significant.

## Results

### ALF-186 treatment protects retinal ganglion cells against ischemia reperfusion injury (IRI) in a time-dependent manner

**Fig 2:**
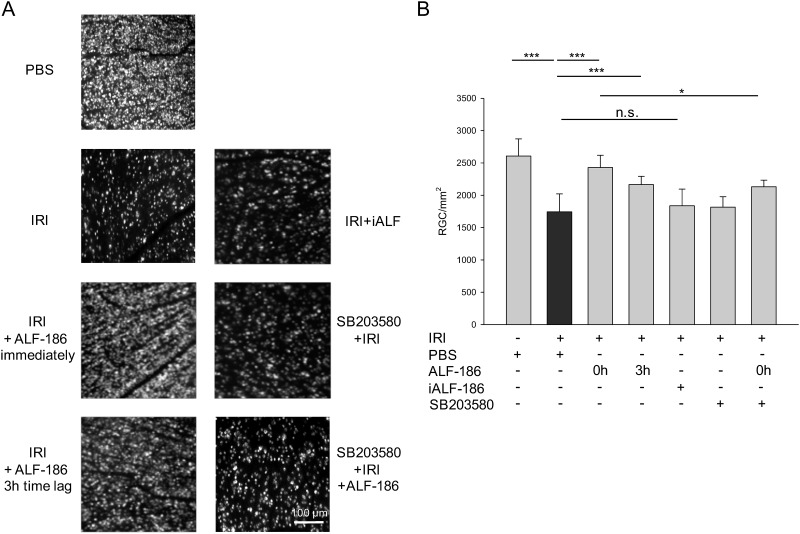
Effect of ALF-186 on vital retinal ganglion cells after ischemia reperfusion injury (IRI) in vivo. (A) Representative flat mount images (n = 8) of fluorogold-labeled retinal ganglion cells 7 days after IRI, with immediate (0 h) and delayed (3 h) ALF-186 treatment, inactivated ALF-186 treatment and p38 inhibition using SB203580. Scale bar is 100 μm. (B) Quantification of retinal ganglion cell density (cells/mm^2^; data are mean±SD; n = 8; IRI vs. IRI+ALF-186 0h and 3h, *** = p<0.001, IRI+ALF-186 0h vs. IRI+ALF-186+SB203580, * = p<0.05).

To answer the question of the influence of ALF-186 regarding retinal ganglion cell survival, the amount of vital neuronal cells was quantified. Retinal IRI damage caused a loss of retinal ganglion cells of approximately 40% (Fig 2A respective image and Fig 2B: columns 1 and 2: IRI 1808±262 vs. untreated 2503±295; *** = p<0.001). Immediate (0 h) treatment with ALF-186 almost completely neutralized this effect (Fig 2A and 2B, column 3: IRI 1808±262 vs. IRI+ALF-186 0 h 2499±377; *** = p<0.001). Even delayed ALF-186 application (3 h) reduced RGC loss significantly (IRI 1808±262 vs. IRI±ALF-186 3h 2202±239; p*** = <0.001). In contrast, administration of inactivated ALF-186 (iALF) did not result in a change of RGC compared to untreated animals. The p38 inhibitor SB203580, injected prior to retinal IRI, abolished ALF´s effect in part (Fig 2A and 2B, column 7: IRI+ALF-186 2499±377 vs. SB203580+IRI+ALF-186 2133±101; * = p<0.05). SB203580 alone showed no effect on ganglion cell loss (IRI 1808±262 vs. IRI+SB203580 1816±162).

### ALF-186 suppresses phosphorylation of ERK1/2, while inducing phosphorylation of p38 after IRI

**Fig 3:**
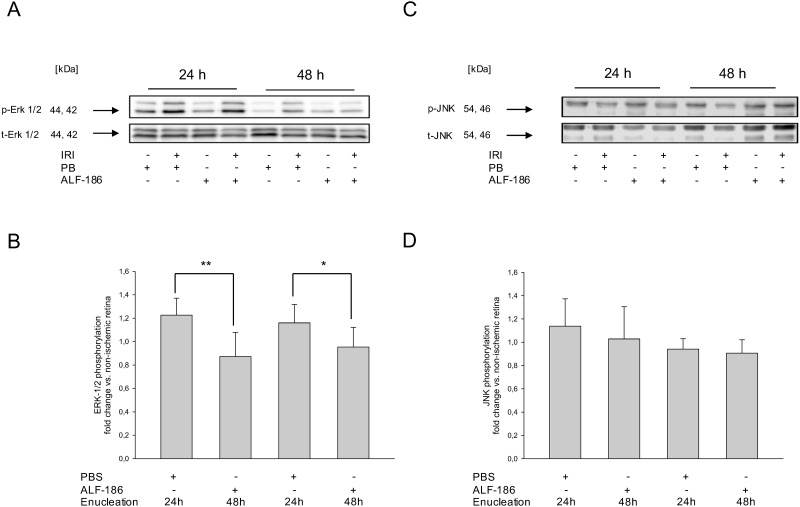
Effect of ALF-186 treatment on the phosphorylation of the mitogen-activated protein (MAP) kinases ERK1/2 and JNK after IRI. (A) Representative western blot image (n = 8) showing the suppression of the phosphorylated MAPK ERK1/2 compared to total ERK1/2 after immediate ALF-186 treatment. Enucleation was either performed 24 hours or 48 hours after IRI. (B) Densitometric analysis (n = 8) of western blots for phosphorylated ERK1/2 compared to its total protein formation 24 and 48 hours after IRI+ALF-186 (data are mean±SD; IRI vs. IRI+ALF-186 24h, ** = p<0.01 and IRI vs. IRI+ALF-186 48h, * = p<0.05). (C) Representative western blot image (n = 8) showing no effect of the phosphorylated MAPK JNK compared to total JNK after immediate ALF-186 treatment. Enucleation was either performed 24 hours or 48 hours after IRI. (D) Densitometric analysis (n = 8) of western blots for phosphorylated JNK compared to its total protein formation 24 and 48 hours after IRI+ALF-186.

To answer the question if intracellular signaling mechanisms of apoptosis are involved, we analyzed the effect of ALF-186 on the phosphorylation of MAPK. After IRI, treatment with ALF-186 decreased IRI-induced ERK1/2 phosphorylation. The extent depended on the time-point of enucleation, with a stronger effect at 24 than 48 hours after IRI (Fig 3A and 3B; IRI 1.23±0.14 vs. IRI+ALF-186 0.87±0.21 fold change at 24 h; ** = p<0.01; IRI 1.16±0.16 vs. IRI+ALF-186 0.95±0.17 fold change at 48 h; * = p<0.05). In contrast, no ALF-186 mediated effect was detectable for JNK-phosphorylation (Fig 3C and 3D; IRI 1.14±0.23 vs. IRI+ALF-186 1.03±0.28 fold change at 24 h; IRI 0.94±0.09 vs. IRI+ALF-186 0.90±0.12 fold change at 48 h).

**Fig 4:**
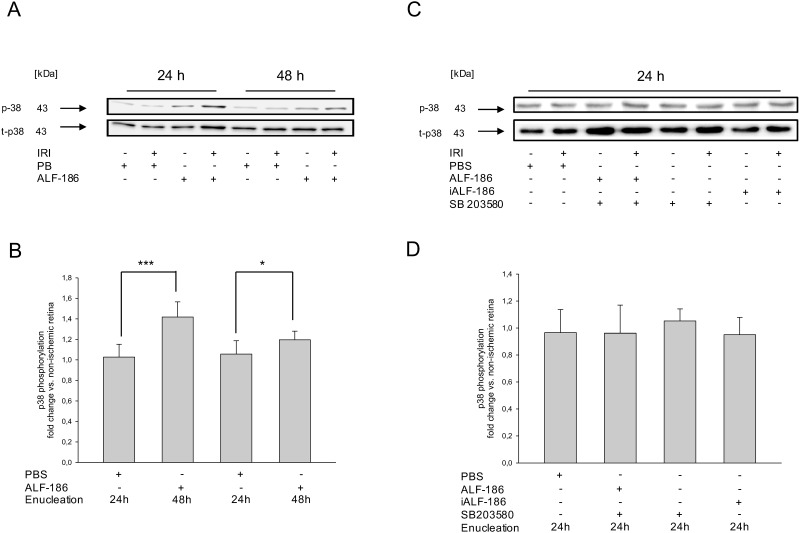
Effect of ALF-186 treatment on the phosphorylation of the MAP kinase p38 after IRI. (A) Representative western blot image (n = 8) showing the increase of phosphorylated p38 compared to total p38 after immediate ALF-186 treatment. Enucleation was either performed 24 hours or 48 hours post IRI. (B) Densitometric analysis of n = 8 western blots for phosphorylated p38 compared to its total protein formation after IRI+ALF-186 (data are mean±SD; IRI vs. IRI+ALF-186 24h, *** = p<0.001 and IRI vs. IRI+ALF-186 48h, * = p<0.05). (C) Representative western blot image (n = 8) showing the abolishment of p38 phosphorylation due to either iALF-186 treatment or inhibition of p38 using SB203580 compared to total p38 after immediate ALF-186 treatment. Enucleation was performed 24 hours post IRI. (D) Densitometric analysis of n = 8 western blots for phosphorylated p38 compared to its total protein formation after IRI and respective treatment.

Instead, ALF-186 significantly induced p38-phosphorylation. Again, we observed a more pronounced effect at 24 h after IRI (Fig 4A and 4B; IRI 1.03±0.12 vs. IRI+ALF-186 1.42±0.15 fold change at 24 h; *** = p<0.001; IRI 1.06±0.13 vs. IRI+ALF-186±1.20±0.08 fold change at 48 h; * = p<0.05). Inactivated ALF-186 did not affect p38 phosphorylation (Fig 4C and 4D; IRI 1.05±0.09 vs. IRI+iALF-186 0.95±0.13). To confirm ALF´s mechanism of action regarding the involvement of p38, we next injected the p38 inhibitor SB203580 intravenously prior to IRI. While SB203580 had no influence on p38-phosphorylation after IRI, its presence significantly reduced phosphorylation of p38 in the context of ALF-186. This effect was quantified densitometrically, showing that SB203580 abolished the ALF-186 mediated increase of p38-phosphorylation completely (Fig 4C and 4D; IRI 0.90±0.35 vs. SB203580+IRI+ALF-186 0.90±0.32).

### ALF-186 inhibits IRI-induced Caspase-3 mRNA expression and Cleavage

To study the extent to which ALF-186 influences apoptosis, we next analyzed Caspase-3 mRNA expression and cleavage.

**Fig 5:**
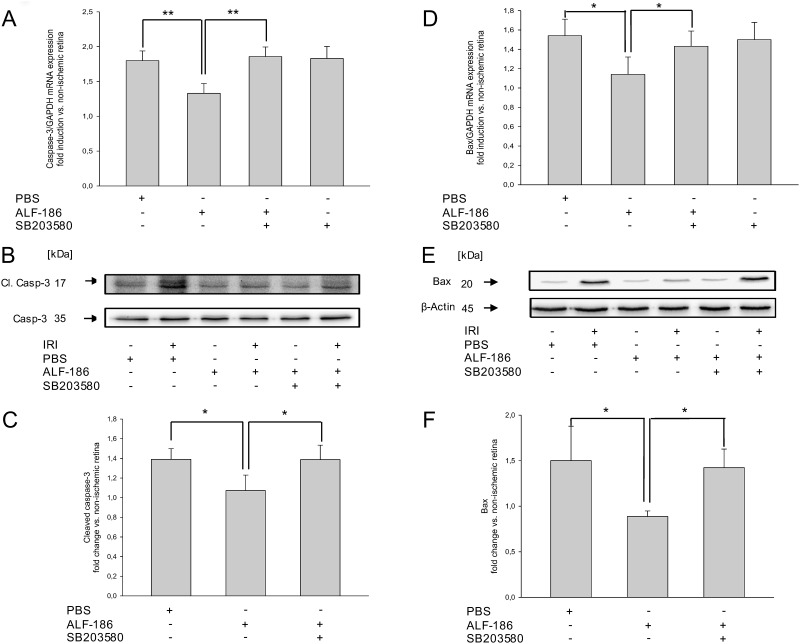
Effect of ALF-186 treatment on retinal mRNA expression and protein cleavage of Caspase-3 and protein and mRNA expression of Bax. (A) Fold induction of Caspase-3 mRNA after IRI+ALF-186 and p38 inhibition in ischemic retinal tissue compared to GAPDH in relation to the corresponding non-ischemic retinae analyzed by RT-PCR (n = 8; data are mean±SD; IRI vs. IRI+ALF-186, ** = p<0.01 and IRI+ALF-186 vs. SB203580+IRI+ALF-186, ** = p<0.01). (B) Representative western blot image showing the induction of caspase-3 cleavage due to IRI (lane 2) and its suppression (lane 4) compared to uncleaved caspase-3 after ALF-186 treatment. This effect was abrogated by p38 inhibition using SB203580 (lane 6). (C) Densitometric analysis of n = 8 western blots for Caspase-3 cleavage (data are mean±SD; IRI vs. IRI+ALF-186, * = p<0.05 and IRI+ALF-186 vs. IRI+SB203580+ALF-186, * = p<0.05). (D) Fold induction of Bax mRNA after IRI+ALF-186 and p38 inhibition in ischemic retinal tissue compared to GAPDH in relation to the corresponding non-ischemic retinae analyzed by RT-PCR (n = 8; data are mean±SD; IRI vs. IRI+ALF-186, * = p<0.05 and IRI+ALF-186 vs. SB203580+IRI+ALF-186, * = p<0.05). (E) Representative western blot image showing the induction of Bax due to IRI (lane 2) and its suppression (lane 4) compared to β-Actin after ALF-186 treatment. This effect was abrogated by p38 inhibition using SB203580 (lane 6). (F) Densitometric analysis of n = 8 western blots for Bax expression (data are mean±SD; IRI vs. IRI+ALF-186, * = p<0.05 and IRI+ALF-186 vs. SB203580+IRI+ ALF-186, * = p<0.05).

ALF-186 treatment decreased IRI-induced Caspase-3 mRNA expression significantly (Fig 5A; IRI 1.80±0.14 vs. IRI+ALF-186 1.33±0.14; ** = p<0.01). P38 inhibition prior to IRI and ALF-186 treatment abrogated ALF-186 mediated reduction of Caspase-3 mRNA (Fig 5A; IRI+ALF-186 1.33±0.14 vs. IRI+SB203580+ALF-186 1.86±0.14; ** = p<0.01), while the inhibitor alone had no effect.

ALF-186 effects Caspase-3 cleavage in a similar way. Animals treated with ALF-186 showed reduced Caspase-3 cleavage after IRI (Fig 5B and 5C; IRI 1.39±0.11 vs. IRI+ALF-186 1.07±0.16; * = p<0.05). SB203580 significantly reduced ALF´s effect (Fig 5B, 1.07±0.16 vs. 1.39±0.15; * = p<0.05).

### ALF-186 reduces mRNA and protein expression of Bax

To further analyze the role of ALF-186 in apoptosis, we evaluated the effect of ALF-186 on Bax, a protein enhancing apoptosis. Treatment with ALF-186 significantly decreased IRI induced Bax mRNA (Fig 5D; IRI 1.54±0.17 vs. IRI+ALF-186 1.14±0.18; * = p<0.05) as well as protein expression (Fig 5E; IRI 1.50±0.38 vs. IRI+ALF-186 0.89±0.06; * = p<0.05). P38 inhibition prior to IRI and ALF-186 treatment significantly increased Bax mRNA and protein expression again (Fig 5D; IRI+ALF-186 0.89±0.06 vs. SB203580+IRI+ALF-186 1.43±0.16; * = p<0.05; Fig 5F; IRI+ALF-186 0.89±0.06 vs. SB203580+IRI+ALF-186 1.42±0.20; * = p<0.05), while the inhibitor alone had no effect.

### ALF-186 increases mRNA and protein expression of Bcl-2

**Fig 6:**
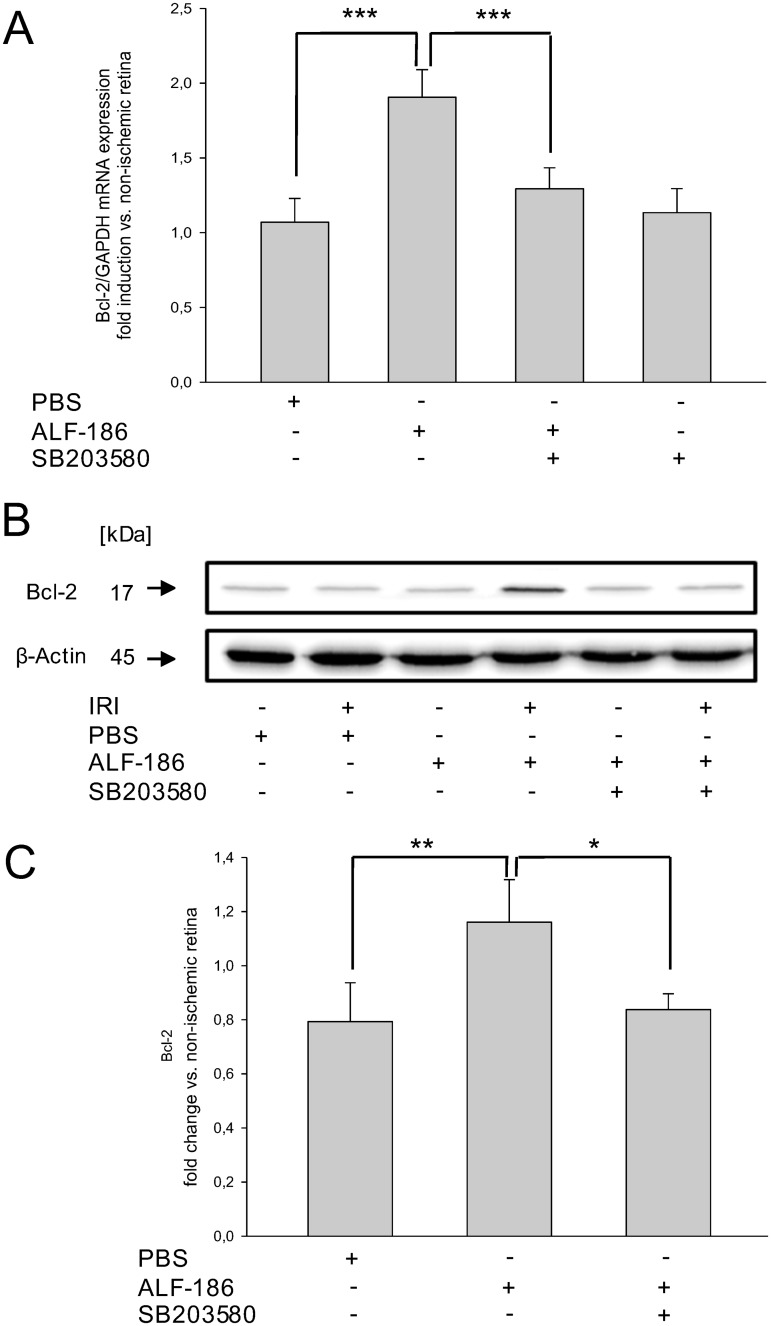
Effect of ALF-186 treatment on retinal protein and mRNA expression of Bcl-2. (A) Fold induction of Bcl-2 mRNA after IRI+ALF-186 and p38 inhibition in ischemic retinal tissue compared to GAPDH in relation to the corresponding non-ischemic retinae analyzed by RT-PCR (n = 8; data are mean±SD; IRI vs. IRI+ALF-186, *** = p<0.001 and IRI+ALF-186 vs. SB203580+IRI+ALF-186, *** = p<0.001). (E) Representative western blot image showing no change of Bcl-2 due to IRI (lane 2) but its strong induction (lane 4) compared to β-Actin after ALF-186 treatment. This effect was abrogated by p38 inhibition using SB203580 (lane 6). (F) Densitometric analysis of n = 8 western blots for Bax expression (data are mean±SD; IRI vs. IRI+ALF-186, ** = p<0.01 and IRI+ALF-186 vs. SB203580+IRI+ ALF-186, * = p<0.05).

Moreover, Bcl-2, a protein known to inhibit apoptosis, was analyzed to answer the question of ALF-186 influence on apoptosis. ALF-186 treatment significantly increased Bcl-2 mRNA (Fig 6A; IRI 1.07±0.16 vs. IRI+ALF-186 1.91±0.18; *** = p<0.001) and protein expression (Fig 6B and 6C; n = 6; IRI 0.79±0.14 vs. IRI+ALF-186 1.16±0.16; ** = p<0.01) after IRI. This effect was reduced significantly by p38 inhibition (Fig 6A; IRI+ALF-186 1.91±0.18 vs. SB203085+IRI+ALF-186 1.29±0.14, *** = p<0.001; Fig 6B and 6C; IRI+ALF-186 1.16±0.16 vs. SB203085+IRI+ALF-186 0.84±0.06; * = p<0.05), while the inhibitor alone had no effect.

### ALF-186 induced p38 expression in the ganglion cell layer and inner nuclear layer

**Fig 7:**
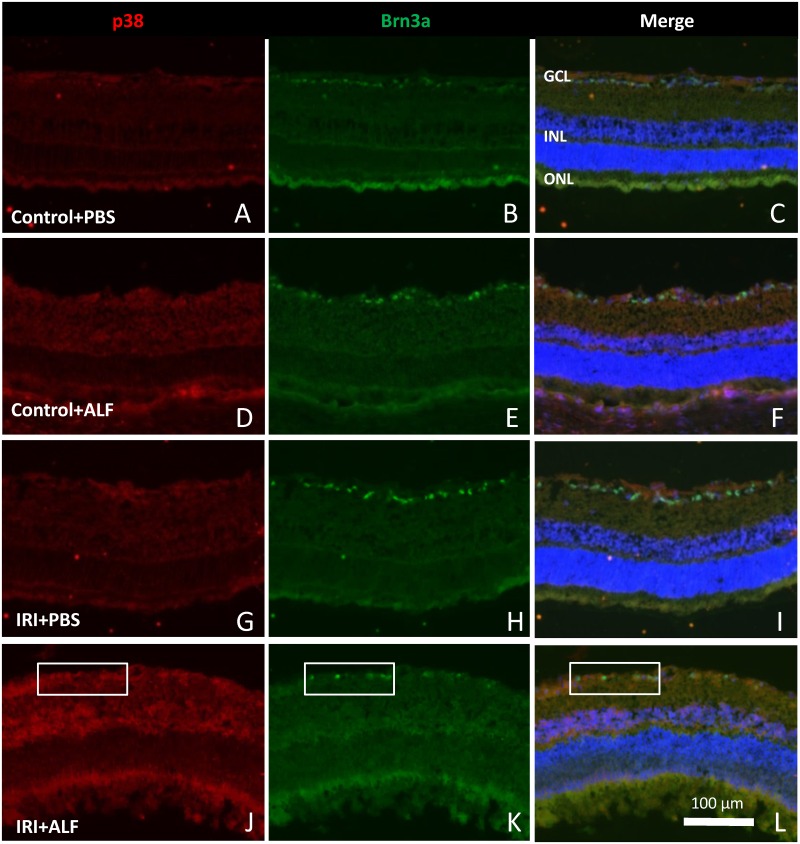
ALF-186 induced p38 MAPK in retinal ganglion cell layer. Retinal cross sections were evaluated for p38 immunoreactivity to answer the question, which cells were responsible for p38 upregulation after IRI+ALF. In controls and after IRI+PBS, there was only a weak p38 expression, pronounced in the area of the ganglion cell layer (GCL) (Fig 5, right side, A, D, G). Co-staining with Brn3a (B, E, H) revealed a low degree of double-immunoreactivity, the p38 signal was seen in a low level in the area of the nerve fibers on top of the GCL (C, F, I). ALF treatment directly after IRI induced upregulation of p38 (J) in the GCL and inner nuclear layer (INL), as expected from the results of the p38 mRNA expression. Double-immunoreactivity with Brn3a (K+L) was more frequent and more intense but not exclusively expressed in RGCs. Scale bar in L is 100 μm.

**Fig 8:**
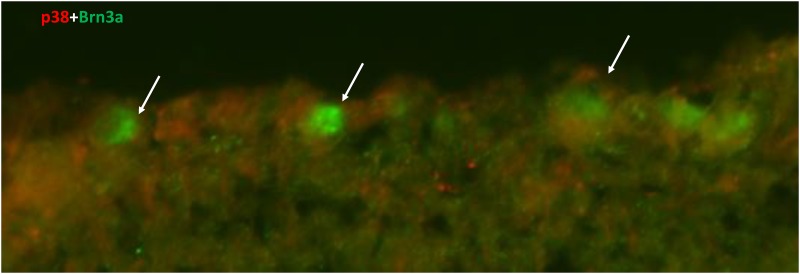
Double staining of p38 and Brn3a. Magnification of the GCL of Fig 7, picture L; demonstrating the double staining of p38 and Brn3a. The arrow points to a double stained retinal ganglion cell.

Retinal cross sections were evaluated for p38 immunoreactivity to answer the question, which cells were responsible for p38 upregulation after IRI+ALF. In untreated eyes and after IRI, there was only a weak p38 expression pronounced in the area of the ganglion cell layer (GCL) (Fig 7A, 7D and 7G). Co-staining with Brn3a (B, E, H) revealed a low degree of double-immunoreactivity with RGCs, the p38 signal was seen in a low level in the area of the nerve fibers on top of the GCL (C, F, I). ALF treatment directly after IRI induced upregulation of p38 (J) in the GCL and inner nuclear layer (INL). Double-immunoreactivity with Brn3a (L and Fig 8) was partially seen. Scale bar in L is 100 μm.

## Discussion

The main findings of this *in vivo* study can be summarized as follows: (1) Treatment with the carbon monoxide releasing molecule (CORM) ALF-186 alleviated loss of retinal ganglion cells due to ischemia reperfusion injury (IRI). (2) ALF-186 mediates anti-apoptotic signaling via p38, but not extracellular regulated kinases (ERK1/2) and cJun N-terminal kinases (JNK). (3) ALF-186 mediated anti-apoptotic signaling, decreasing caspase-3 and the pro-apoptotic protein Bax, while increasing anti-apoptotic proteins like Bcl-2. (4) Inhibition of p38 MAPK counteracted ALF´s protective effect in retinal IRI. (5) ALF´s effect on molecular mechanisms predominantly proceeded within the first 24h showing a marked fading thereafter.

Carbon monoxide (CO) exerts a variety of pharmacological effects, mediating among others anti-apoptotic effects in the cardiovascular, immune and nervous systems.[20, 21] Due to difficult handling in treatment with the potentially toxic volatile agent CO, carbon monoxide releasing molecules have attracted attention. These molecules contain metal carbonyls like manganese, ruthenium, boron and iron as CO carrier leading to a constant and reliable release of CO.^1^, [15] Since water-soluble substances are available, oral or intravenous application simplified CO treatment avoiding environmental impact and potential poisoning.

In this *in-vivo* study, we have examined the potential protective effect of ALF-186 on neuronal tissue. Hence, we chose a well-established model of retinal IRI in rats.[17–19] We found that treatment with ALF-186 reduced IRI mediated loss of retinal ganglion cells and therefore mediated protection. While protective effects of carbon monoxide are well studied in a variety of damage models of non-neuronal organs, studies describing the protective effect of CO and CORM on neuronal tissue are limited.[22–26] Zeynalov and Doré demonstrated that inhalative carbon monoxide reduced infarct size after focal transient brain ischemia in a mouse model.[27] Wang et al. also exposed mice to carbon monoxide after middle cerebral artery occlusion and described a reduced infarct volume.[28] The neuroprotective effects in this study are comparable to our previous findings.[17]

Intracellular signaling via MAPK ERK1/2, p38 and JNK plays a significant role in the regulation of neuronal [29–31] and retinal [32–34] apoptosis. However, the effects of ERK1/2 phosphorylation are considered to be contradictory: both survival and death signals can activate ERK1/2 and therefore activation itself may result in neuronal death or survival.[29] Cellular stress triggers MAPK p38 and JNK activation, for example in the context of cerebral ischemia.[35] It is assumed that p38 and JNK mediated apoptosis is transmitted by mitochondrial pro-apoptotic proteins like Bax and Bcl-2.[35]

In our study we have found that ALF 186 modulated MAPK differently. ALF treatment resulted in a decreased ERK1/2 phosphorylation and, on contrary, increased p38 activation while JNK phosphorylation did not changed. Following cell and tissue damage, the influence of carbon monoxide on MAPK is a topic of controversy in the literature whereby it is important to note that the type of injury differently affected the MAPK. Our findings are in accordance to the results of Sethi et al., who exposed primary pulmonary artery endothelial cells of the rat to carbon monoxide after TNFα stimulation. Injured cells presented increased ERK1/2 and JNK activation in contrast to p38 suppression. Application of CO reversed TNFα mediated effects on ERK1/2 and p38 while CO had no influence on JNK.[36] Li et al. describe in their study the p38 MAPK pathway as a key signaling mechanism of CO´s protective effect. In primary hepatocytes of rats´ ethanol increased p38 phosphorylation while additional CORM further stimulated p38 phosphorylation. JNK and ERK1/2 were not affected.[37] The cytoprotective effect of ERK1/2 downregulation was described by Choi et al. Glucose deprivation led to an increase of ERK1/2 and p38 phosphorylation in BNL CL.2 cells whereas JNK was not affected. In contrast to our findings, the presence of CORM only suppressed ERK1/2 phosphorylation.[38] Ning et al. showed in their *in-vitro* study that stimulating lung cells, with IL16 only affected MAPK ERK1/2, but neither p38 nor JNK, and increased levels of phosphorylated ERK1/2. Exposure to CO (250 ppm) mitigated this effect.[39] Similarly, CO treatment suppressed elevated ERK1/2 phosphorylation in liver grafts after cold IRI due to orthotopic liver transplantation in rats. Although CO also decreased p38 phosphorylation, the influence of CO was identified as not significant.[13] Our research group showed that CORM increased p38 phosphorylation in staurosporine treated EBL cells, but not ERK1/2 nor JNK.[40] In another study, postconditioning with inhalative CO (250 ppm) showed similar effects on retinal ganglion cells with an increase of cell count after IRI. Interestingly, an opposite effect on MAPK was demonstrated.[19] Inhalative CO reduced p38 phosphorylation and induced ERK1/2 activation while JNK was not affected. The reason for this might be the different way of administration.

Due to neuronal injury, neurons release molecules interacting cell survival and cell death signaling. In particular, Bcl-2 family proteins like Bax and Bcl-2 are involved in IRI.[41] Thereby, Bcl-2 promotes neuronal survival modulating Ca^2+^ signaling and Bax operates as an apoptotic stimulus. Shifting this balanced Bax/Bcl-2 ratio towards Bax causes mitochondrial damage that activates the caspase cascade.[42]

Our study demonstrated, that ALF-186 reduced IRI induced apoptosis by inhibition of Bax expression, activation of Bcl-2 and, consecutively, by suppression of caspase-3 cleavage. In part, these results are in agreement with Schallner et al.: Inhalation of CO prior to IRI caused a decrease of Bax and caspase-3 cleavage while Bcl-2 was not affected.[19] Cheng and Levy also reported a beneficial effect of carbon monoxide on neuronal tissue. In their study, subclinical CO decreased caspase-3 activation in mice pulps following isoflurane exposition.[43] In a study with endothelial lung cells Wang et al. described that CO inhibited hyperoxia induced cell death by decreasing pro-apoptotic Bax.[44] Nakao et al. examined the effect of CO treatment on intestinal apoptosis due to IRI in rats. CO inhalation reduced Bax and upregulated Bcl-2.[45]

**Fig 9:**
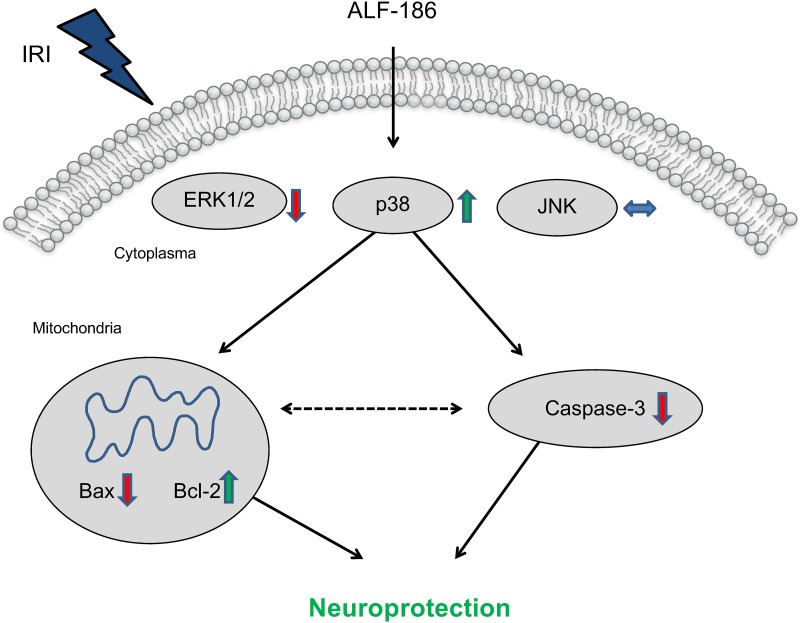
Diagram showing the proposed mechanism of ALF-186 mediated anti-apoptotic effect on retinal ganglion cells. ALF treatment affects mitogen-activated protein kinases ERK1/2 and p38. Due to increased p38 phosphorylation ALF-186 reduced pro-apoptotic Bax protein expression and caspase-3 cleavage while increasing anti-apoptotic Bcl-2, thus promoting neuroprotection.

Pretreatment with p38 inhibitor SB203580 reduced the impact of ALF-186 on retinal apoptosis. We have found that p38 inhibition resulted in an increase of caspase-3 cleavage and Bax expression and also in a decrease of Bcl-2 expression. Furthermore, p38 inhibition reversed ALF-186 mediated p38 phosphorylation, reduced neuronal ganglion cell count and cancelled neuroprotection. We focused on this proposed mechanism as part of the carbon monoxide mediated neuroprotective effect in Fig 9. Although neuroprotective effects are described [46], in our study SB203580 alone did not affect retinal apoptosis. This is also confirmed by Mori et al., who did not notice protection in traumatic brain injury after treatment with SB203580.[47]

In summary, we demonstrate that the treatment with ALF-186 as a therapeutic option reduced retinal apoptosis after IRI *in-vivo* mainly in the first 24 hours after neuronal damage. ALF186 affected and modulated intracellular MAPK and apoptosis signaling and reduced loss of retinal ganglion cells, therefore representing a novel and promising alternative in treating ischemia-related injury of neuronal organs.

## References

[1] Abe K, Aoki M, Kawagoe J, Yoshida T, Hattori A, Kogure K, et al Ischemic delayed neuronal death. A mitochondrial hypothesis. Stroke. 1995;26(8):1478–89. 7631357 10.1161/01.str.26.8.1478

[2] Schmidt-Kastner R, Freund TF. Selective vulnerability of the hippocampus in brain ischemia. Neuroscience. 1991;40(3):599–636. 1676492 10.1016/0306-4522(91)90001-5

[3] Mortier E, Struys M, Herregods L. Therapeutic coma or neuroprotection by anaesthetics. Acta neurologica Belgica. 2000;100(4):225–8. 11233677

[4] Donnan GA, Fisher M, Macleod M, Davis SM. Stroke. Lancet. 2008;371(9624):1612–23. 10.1016/S0140-6736(08)60694-7 18468545

[5] Verma D. Pathogenesis of diabetic retinopathy—the missing link? Medical hypotheses. 1993;41(3):205–10. 7505046 10.1016/0306-9877(93)90231-e

[6] Nickells RW. Retinal ganglion cell death in glaucoma: the how, the why, and the maybe. Journal of glaucoma. 1996;5(5):345–56. 8897235

[7] Moretti A, Ferrari F, Villa RF. Neuroprotection for ischaemic stroke: current status and challenges. Pharmacology & therapeutics. 2015;146:23–34. 25196155 10.1016/j.pharmthera.2014.09.003

[8] Wu L, Wang R. Carbon monoxide: endogenous production, physiological functions, and pharmacological applications. Pharmacological reviews. 2005;57(4):585–630. 10.1124/pr.57.4.3 16382109

[9] Ryter SW, Otterbein LE. Carbon monoxide in biology and medicine. BioEssays: news and reviews in molecular, cellular and developmental biology. 2004;26(3):270–80. 10.1002/bies.20005 14988928

[10] Kohmoto J, Nakao A, Kaizu T, Tsung A, Ikeda A, Tomiyama K, et al Low-dose carbon monoxide inhalation prevents ischemia/reperfusion injury of transplanted rat lung grafts. Surgery. 2006;140(2):179–85. 10.1016/j.surg.2006.03.004 16904967

[11] Li Volti G, Rodella LF, Di Giacomo C, Rezzani R, Bianchi R, Borsani E, et al Role of carbon monoxide and biliverdin in renal ischemia/reperfusion injury. Nephron Experimental nephrology. 2006;104(4):e135–9. 10.1159/000094964 16902317

[12] Nakao A, Toyokawa H, Abe M, Kiyomoto T, Nakahira K, Choi AM, et al Heart allograft protection with low-dose carbon monoxide inhalation: effects on inflammatory mediators and alloreactive T-cell responses. Transplantation. 2006;81(2):220–30. 10.1097/01.tp.0000188637.80695.7f 16436966

[13] Kaizu T, Ikeda A, Nakao A, Tsung A, Toyokawa H, Ueki S, et al Protection of transplant-induced hepatic ischemia/reperfusion injury with carbon monoxide via MEK/ERK1/2 pathway downregulation. American journal of physiology Gastrointestinal and liver physiology. 2008;294(1):G236–44. 10.1152/ajpgi.00144.2007 18006605

[14] Jasnos K, Magierowski M, Kwiecien S, Brzozowski T. [Carbon monoxide in human physiology—its role in the gastrointestinal tract]. Postepy higieny i medycyny doswiadczalnej (Online). 2014;68:101–9. 24491901 10.5604/17322693.1087527

[15] Motterlini R, Clark JE, Foresti R, Sarathchandra P, Mann BE, Green CJ. Carbon monoxide-releasing molecules: characterization of biochemical and vascular activities. Circulation research. 2002;90(2):E17–24. 11834719 10.1161/hh0202.104530

[16] Motterlini R, Otterbein LE. The therapeutic potential of carbon monoxide. Nature reviews Drug discovery. 2010;9(9):728–43. 10.1038/nrd3228 20811383

[17] Biermann J, Lagreze WA, Dimitriu C, Stoykow C, Goebel U. Preconditioning with inhalative carbon monoxide protects rat retinal ganglion cells from ischemia/reperfusion injury. Investigative ophthalmology & visual science. 2010;51(7):3784–91. 20181836 10.1167/iovs.09-4894

[18] Biermann J, Lagreze WA, Schallner N, Schwer CI, Goebel U. Inhalative preconditioning with hydrogen sulfide attenuated apoptosis after retinal ischemia/reperfusion injury. Molecular vision. 2011;17:1275–86. 21633713 PMC3103742

[19] Schallner N, Fuchs M, Schwer CI, Loop T, Buerkle H, Lagreze WA, et al Postconditioning with inhaled carbon monoxide counteracts apoptosis and neuroinflammation in the ischemic rat retina. PloS one. 2012;7(9):e46479 10.1371/journal.pone.0046479 23029526 PMC3460901

[20] Motterlini R, Mann BE, Foresti R. Therapeutic applications of carbon monoxide-releasing molecules. Expert opinion on investigational drugs. 2005;14(11):1305–18. 10.1517/13543784.14.11.1305 16255672

[21] Motterlini R, Mann BE, Johnson TR, Clark JE, Foresti R, Green CJ. Bioactivity and pharmacological actions of carbon monoxide-releasing molecules. Current pharmaceutical design. 2003;9(30):2525–39. 14529551 10.2174/1381612033453785

[22] Neto JS, Nakao A, Kimizuka K, Romanosky AJ, Stolz DB, Uchiyama T, et al Protection of transplant-induced renal ischemia-reperfusion injury with carbon monoxide. American journal of physiology Renal physiology. 2004;287(5):F979–89. 10.1152/ajprenal.00158.2004 15292046

[23] Schwer CI, Mutschler M, Stoll P, Goebel U, Humar M, Hoetzel A, et al Carbon monoxide releasing molecule-2 inhibits pancreatic stellate cell proliferation by activating p38 mitogen-activated protein kinase/heme oxygenase-1 signaling. Molecular pharmacology. 2010;77(4):660–9. 10.1124/mol.109.059519 20053955

[24] Otterbein LE, Mantell LL, Choi AM. Carbon monoxide provides protection against hyperoxic lung injury. The American journal of physiology. 1999;276(4 Pt 1):L688–94. 10198367 10.1152/ajplung.1999.276.4.L688

[25] Fujimoto H, Ohno M, Ayabe S, Kobayashi H, Ishizaka N, Kimura H, et al Carbon monoxide protects against cardiac ischemia—reperfusion injury in vivo via MAPK and Akt—eNOS pathways. Arteriosclerosis, thrombosis, and vascular biology. 2004;24(10):1848–53. 10.1161/01.ATV.0000142364.85911.0e 15308554

[26] Nakao A, Kaczorowski DJ, Sugimoto R, Billiar TR, McCurry KR. Application of heme oxygenase-1, carbon monoxide and biliverdin for the prevention of intestinal ischemia/reperfusion injury. Journal of clinical biochemistry and nutrition. 2008;42(2):78–88. 10.3164/jcbn.2008013 18385824 PMC2266059

[27] Zeynalov E, Dore S. Low doses of carbon monoxide protect against experimental focal brain ischemia. Neurotoxicity research. 2009;15(2):133–7. 10.1007/s12640-009-9014-4 19384576 PMC2719876

[28] Wang B, Cao W, Biswal S, Dore S. Carbon monoxide-activated Nrf2 pathway leads to protection against permanent focal cerebral ischemia. Stroke. 2011;42(9):2605–10. 10.1161/STROKEAHA.110.607101 21852618 PMC3278075

[29] Subramaniam S, Unsicker K. ERK and cell death: ERK1/2 in neuronal death. The FEBS journal. 2010;277(1):22–9. 10.1111/j.1742-4658.2009.07367.x 19843173

[30] Takeda K, Ichijo H. Neuronal p38 MAPK signalling: an emerging regulator of cell fate and function in the nervous system. Genes to cells: devoted to molecular & cellular mechanisms. 2002;7(11):1099–111. 12390245 10.1046/j.1365-2443.2002.00591.x

[31] Antoniou X, Borsello T. The JNK signalling transduction pathway in the brain. Frontiers in bioscience (Elite edition). 2012;4:2110–20. 22202023 10.2741/e528

[32] Jiang SY, Zou YY, Wang JT. p38 mitogen-activated protein kinase-induced nuclear factor kappa-light-chain-enhancer of activated B cell activity is required for neuroprotection in retinal ischemia/reperfusion injury. Molecular vision. 2012;18:2096–106. 22876136 PMC3413424

[33] Schallenberg M, Charalambous P, Thanos S. GM-CSF regulates the ERK1/2 pathways and protects injured retinal ganglion cells from induced death. Experimental eye research. 2009;89(5):665–77. 10.1016/j.exer.2009.06.008 19560459

[34] Kim BJ, Silverman SM, Liu Y, Wordinger RJ, Pang IH, Clark AF. In vitro and in vivo neuroprotective effects of cJun N-terminal kinase inhibitors on retinal ganglion cells. Molecular neurodegeneration. 2016;11:30 10.1186/s13024-016-0093-4 27098079 PMC4839164

[35] Mehta SL, Manhas N, Raghubir R. Molecular targets in cerebral ischemia for developing novel therapeutics. Brain research reviews. 2007;54(1):34–66. 10.1016/j.brainresrev.2006.11.003 17222914

[36] Sethi JM, Otterbein LE, Choi AM. Differential modulation by exogenous carbon monoxide of TNF-alpha stimulated mitogen-activated protein kinases in rat pulmonary artery endothelial cells. Antioxidants & redox signaling. 2002;4(2):241–8. 12006175 10.1089/152308602753666299

[37] Li Y, Gao C, Shi Y, Tang Y, Liu L, Xiong T, et al Carbon monoxide alleviates ethanol-induced oxidative damage and inflammatory stress through activating p38 MAPK pathway. Toxicology and applied pharmacology. 2013;273(1):53–8. 10.1016/j.taap.2013.08.019 23994557

[38] Choi BM, Pae HO, Kim YM, Chung HT. Nitric oxide-mediated cytoprotection of hepatocytes from glucose deprivation-induced cytotoxicity: involvement of heme oxygenase-1. Hepatology (Baltimore, Md). 2003;37(4):810–23. 10.1053/jhep.2003.50114 12668974

[39] Ning W, Choi AM, Li C. Carbon monoxide inhibits IL-17-induced IL-6 production through the MAPK pathway in human pulmonary epithelial cells. American journal of physiology Lung cellular and molecular physiology. 2005;289(2):L268–73. 10.1152/ajplung.00168.2004 16003000

[40] Schallner N, Schwemmers S, Schwer CI, Froehlich C, Stoll P, Humar M, et al p38beta-regulated induction of the heat shock response by carbon monoxide releasing molecule CORM-2 mediates cytoprotection in lung cells in vitro. European journal of pharmacology. 2011;670(1):58–66. 10.1016/j.ejphar.2011.08.028 21925493

[41] Anilkumar U, Prehn JH. Anti-apoptotic BCL-2 family proteins in acute neural injury. Frontiers in cellular neuroscience. 2014;8:281 10.3389/fncel.2014.00281 25324720 PMC4179715

[42] Yuan J, Yankner BA. Apoptosis in the nervous system. Nature. 2000;407(6805):802–9. 10.1038/35037739 11048732

[43] Cheng Y, Levy RJ. Subclinical carbon monoxide limits apoptosis in the developing brain after isoflurane exposure. Anesthesia and analgesia. 2014;118(6):1284–92. 10.1213/ANE.0000000000000030 24413549 PMC4029883

[44] Wang X, Wang Y, Kim HP, Nakahira K, Ryter SW, Choi AM. Carbon monoxide protects against hyperoxia-induced endothelial cell apoptosis by inhibiting reactive oxygen species formation. The Journal of biological chemistry. 2007;282(3):1718–26. 10.1074/jbc.M607610200 17135272

[45] Nakao A, Kimizuka K, Stolz DB, Neto JS, Kaizu T, Choi AM, et al Carbon monoxide inhalation protects rat intestinal grafts from ischemia/reperfusion injury. The American journal of pathology. 2003;163(4):1587–98. 10.1016/S0002-9440(10)63515-8 14507665 PMC1868280

[46] Liu XW, Ji EF, He P, Xing RX, Tian BX, Li XD. Protective effects of the p38 MAPK inhibitor SB203580 on NMDAinduced injury in primary cerebral cortical neurons. Molecular medicine reports. 2014;10(4):1942–8. 10.3892/mmr.2014.2402 25051190

[47] Mori T, Wang X, Jung JC, Sumii T, Singhal AB, Fini ME, et al Mitogen-activated protein kinase inhibition in traumatic brain injury: in vitro and in vivo effects. Journal of cerebral blood flow and metabolism: official journal of the International Society of Cerebral Blood Flow and Metabolism. 2002;22(4):444–52. 10.1097/00004647-200204000-00008 11919515

